# Do multiple community-based interventions on health promotion tackle health inequalities?

**DOI:** 10.1186/s12939-020-01271-8

**Published:** 2020-09-10

**Authors:** Stefan Nickel, Olaf von dem Knesebeck

**Affiliations:** University Medical Center Hamburg-Eppendorf, Institute of Medical Sociology, Martinistraße 52, D-20246 Hamburg, Germany

**Keywords:** Community, Health promotion, Outcome evaluation, SES, Health inequalities

## Abstract

**Background:**

Previous systematic reviews of the impact of multi-component community-based health promotion interventions on reducing health inequalities by socio-economic status (SES) were restricted to physical activity and smoking behavior, and revealed limited and rather disillusioning evidence. Therefore, we conducted a comprehensive review worldwide to close this gap, including a wide range of health outcomes.

**Methods:**

The Pubmed and PsycINFO databases were screened for relevant articles published between January 1999 and August 2019, revealing 87 potentially eligible publications out of 2876 hits. In addition, three studies out of a prior review on the effectiveness of community-based interventions were reanalyzed under the new research question. After a systematic review process, 23 papers met the inclusion criteria and were included in the synthesis.

**Results:**

More than half (56.5%) of the studies reported improvements of socially disadvantaged communities overall (i.e. reduced inequalities at the area level) in at least one health behavior and/or health status outcome. Amongst the remaining studies we found some beneficial effects in the most deprived sub-groups of residents (8.2%) and studies with no differences between intervention and control areas (34.8%). There was no evidence that any program under review resulted in an increase in health disparity.

**Conclusions:**

Our results confirm that community-based interventions may be reducing absolute health inequalities of deprived and disadvantaged populations, but their potential so far is not fully realized. For the future, greater attention should be paid to inequalities between sub-groups within communities when analyzing changes in health inequality over time.

## Introduction

Systematic reviews on the effects of interventions on equity in the field of public health revealed disillusioning and unclear results [1, 2]. Some interventions may reduce, or at least not increase health inequalities, if they are of greater benefit to disadvantaged (higher risk) groups. Such interventions often comprise regulatory measures to improve housing and working conditions as well as economic incentives (e.g. free fruit and veg provision in schools, increase in tobacco tax) [1, 3]. Generally, rather structural, population-related (“upstream”) interventions show these effects. Conversely, a number of researchers have emphasized the danger that public health interventions may increase health inequalities. Where an intervention is of greater benefit to advantaged groups than to others, this can be the case. Typical examples are media campaigns and interventions, which aim at individual education and behavior (“downstream interventions”) or selective risk groups [4, 5].

However, reviews on the impact of these interventions are mixed, raising some concerns about their effects on health inequalities. The underlying socio-ecological framework remains a general theory with no specification of causal pathways, and thus neither identifies specific ways to intervene, nor provides supportive evidence for interventions to reduce inequalities [6, 7]. There are two implications of these findings on reducing health inequalities: For one thing, a ‘one-size-fits-all’ intervention may not be enough, but a combination of multiple intervention strategies is required, such as individual or group education, including broader intervention strategies as environmental changes and policies. Secondly, interventions which are well-tailored for the needs of individuals or sub-groups within a target population may result in better outcomes that are more equitable [8].

In the field of multi-component (“complex”) community-based health promotion interventions there are, to our knowledge, only two reviews from high-income countries which explicitly address inequalities in terms of socio-economic status (i.e. SES including income, occupational status, assets or education), rather than other equity factors such as gender or ethnicity [9, 10]. These reviews focussing on physical activity and smoking behavior have revealed limited evidence for reducing health inequalities. Either there were no differences by SES (education/income; 2 primary studies), or no data was found [1]. Furthermore, a fundamental methodological problem arises in this context: It is possible that interventions improve the health of a population (defined by place of residence or other measures) *overall*, but do not reduce or widen inequalities in health between sub-groups *within* the population due to preferential uptake by the comparatively most advantaged [11, 12].

Thus, the effect on (in)equalities can be classified as follows:

- Intervention likely to reduce inequalities: the intervention preferentially improved health outcomes in people of lower SES.
- Intervention likely to widen inequalities: the intervention preferentially improved health outcomes in people of higher SES.
- Intervention which had no preferential impact by SES (this also includes interventions with an overall benefit but without an effect on health equity between SES sub-groups within a community).

The aim of this paper is to provide a systematic review in order to explore, whether multiple community-based health promotion interventions improve the health of a socially deprived population overall (areal level) and/or reduce inequalities between socio-economically defined sub-groups. Attention will also be paid to the dynamics underlying the observed intervention effects mentioned above, i.e. the inequality within a community may increase while at the same time population health remains constant or even decreases [11].

## Methods

### Protocol

We conducted a systematic review according to the PRISMA-Equity 2012 extension guidelines for systematic reviews with a focus on health equity [13]. An additional checklist shows this in more detail [see Additional File 1].

### Search strategy

**Fig. 1:**
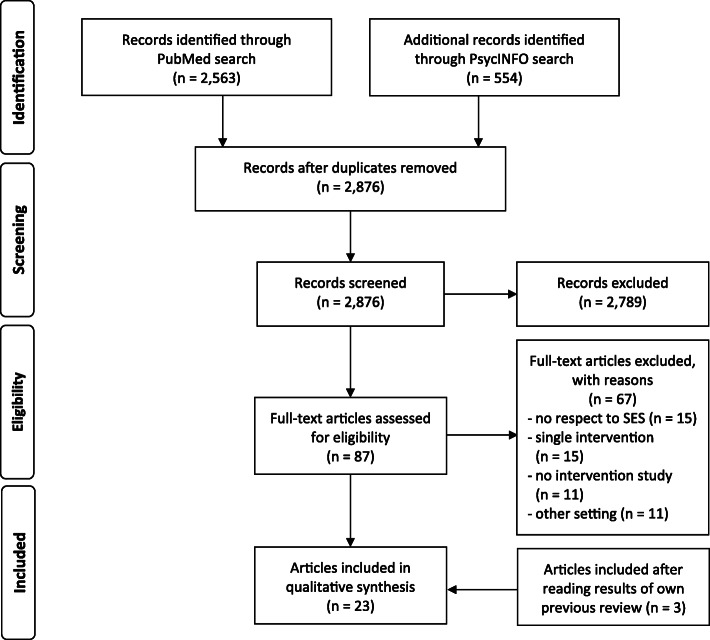
PRISMA flow diagram

The search was limited to articles published in English and German during the period January 1, 1999, to August 31, 2019. The reason for choosing this period of time is that earlier reviews mentioned above [9, 10] are focussed on specific topics and do not contain the more recent studies and newer health promotion strategies; in addition, the present review should complement our prior review on the effectiveness of community-based health promotion interventions in the last 20 years [14]. We searched PubMed and PsycINFO databases (advanced search: title/abstract) using the string “(health promot* OR disease prevention OR intervention*) AND (neighbo$rhood OR communit* OR area* OR district* OR ward* OR urban OR rural) AND (social determinant* OR occupation* OR education* OR socio* status OR income OR SES OR SEP OR social status OR equalit* OR inequalit* OR equit* OR inequit* OR disparit*) AND (effect* OR benefit* OR health outcome* OR impact* OR influence*) AND (randomi$ed OR trial OR quasi-experiment* OR pretest OR posttest OR pre-post OR time series OR controlled stud* OR before and after OR trend OR longitudinal) NOT (clinical OR review OR study protocol)”. PubMed search resulted in 2563 hits, while PsycINFO came to 554 entries; after duplicates were removed 2876 records remained (see Fig. 1). In addition, all included primary studies from our previous review on the effectiveness of community-based interventions were searched [14].

### Selection criteria

We included primary studies which evaluated the effectiveness of complex community-based health promotion interventions in high, middle and low income countries on any health outcome, and which reported differences in intervention effects between SES groups and/or socially deprived populations overall. Table 1 shows all inclusion and exclusion criteria to select eligible studies.

**Table 1:** Inclusion and exclusion criteria

Inclusion	Exclusion
Community	
‚community‘as geographic or political-administrative area (e.g. neighborhood, residential area, district, city, village)	‚community‘as ethnical group (e.g. migration background, religion) or subcultural identity (e.g. gay community) without geographical reference
Article type	
peer-reviewed original articles	editorials, reviews, articles on theory or study design, thesis
Study type and research question	
interventional studies (e.g. RCT, quasi-experimental study, pre-post-test)	observational studies (e.g. cross-sectional or case-control study)
research question on one or more SES factor (e.g. occupation, education, income)	no research question on health inequalities according to SES factors
Intervention	
multiple interventions (two at least)	single interventions
interventions outside the healthcare system offered to people without diagnosed illness	clinical treatments, palliative or rehabilitative interventions

Studies included in the review should refer to the term “community” as a geographically defined area (e.g. neighbourhood, city, village), but not as a whole state or country. Further inclusion criteria were: peer-reviewed articles, intervention studies, assessment of one or more SES factors as well as multi-component strategies of intervention. The latter means that at least two different intervention types were provided, which also reflect social-ecological approaches of health promotion and prevention [7]. This includes changes based on the following six broad types of intervention: 1) social marketing (e.g. campaigns using mass media), 2) individual and group education (e.g. classroom instruction), 3) networking/partnership (e.g. advocacy groups), 4) environmental changes (e.g. greenspaces, availability of healthy food), 5) regulatory interventions (e.g. smoking policies), and 6) improving “sense of community” (e.g. neighborhood parties) [9, 15]. In the literature, these interventions are understood to be interacting components that address different dimensions of complexity and mediating effects, e.g. regarding the outcome, target groups or stakeholders [6].

### Data extraction and synthesis

Screening followed a two-step process with articles filtered by title/abstract, and full text. First author of this review (SN) initially screened the potentially relevant studies. The second author (OK) independently reviewed articles retained for inclusion in the preliminary phase. Full texts of the remaining 87 articles were read and checked by both authors for eligibility, of which 67 were excluded (see Fig. 1). If there were different views, a third colleague was asked to review the article in question (please see Acknowledgement), and a consensus was reached between the authors. Main reasons for exclusion were a missing consideration of SES, single intervention strategies, non-interventional study, and the focus on a non-community setting (e.g. school, workplace). Finally, we extracted data on characteristics of 23 included studies: author and publication date, study design, risk of bias, intervention types, outcome types, and the main findings on health inequalities.

Due to the heterogeneity of the included studies regarding methodological aspects (e.g. target populations, measurement instruments, statistical methods) as well as of the interventions and outcomes no meta-analysis was conducted. However, to identify trends and provide summary statements on intervention-generated inequalities, simple assessments were made for three possible findings by SES: increased inequalities, reduced inequalities, and no difference by SES. Ambivalent results include studies where there was an overall benefit for a disadvantaged population but no effect on health-related outcomes for any SES sub-group within population [8].

## Results

### Description of the studies

The twenty-three studies included in this review were conducted in high or upper-middle income countries (using the World Bank classification). Of these, 14 were set in Europe [16–29], five in North America [30–34], three in Australia/Oceania [35–37], and one study took place in a country in Central America [38] (see Table 2). The studies used various designs, including RCTs (*n* = 1) [38], cluster randomized trials (*n* = 3) [25, 30, 34], quasi-experimental designs (*n* = 11) [16, 18, 22, 23, 27–29, 33, 35–37], pre-post-tests without control group (*n =* 3) [17, 19, 31], and secondary analyses (*n* = 5) [20, 21, 23, 26, 32]. The sample sizes at baseline varied from 200 to nearly 250,000 in 60 intervention areas, with study populations having a variety of socio-demographic and socio-economic backgrounds. Few studies took place in what can be considered rural areas, while the majority of studies was located in urban neighborhoods, districts or cities.

**Table 2:** Findings on inequalities in intervention effect by SES

Author, Year	Study Design and Country	Risk of Bias^**a**^	Intervention Type	Outcome Type	Findings
Abbema et al., 2004 [16]	Quasi-experimental, Netherlands (n of a deprived area: 323; control area I: 322; control area II: 342)	2	1) Social marketing (articles in local newspaper on speeding and safe playing) 2) Individual or group education (e.g. physical exercises, traffic lessons in schools) 3) Networking/partnership (e.g. discussion meetings, professional networking) 4) Environmental change (e.g. dog walking sites, fitting out children’s playground) 5) Regulatory interventions (speed control) 6) Sense of community (multicultural meetings, neighborhood parties)	Perceived health; health-related problems (stress, lack of area safety, parenting problems)	Few reduced inequalities, but even more negative effects (area level)
Bolton et al., 2017 [33]	Quasi-experimental, Australia (n of five areas of socio-economic disadvantage: 2408 children and adolescents, 501 adults; control group: 3163 children and adolescents, 318 adults)	1	1) Social marketing (e.g. newsletters,newspaper articles, project web pages, booklets) 3) Networking/partnership (e.g. partnership agreements, steering committees, school co-ordinator models) 4) Environmental change (e.g. healthy eating in schools, physical activity opportunities)	Anthropometry; health-related behaviors; quality of life	Few reduced inequalities (area level)
Buscail et al., 2016 [17]	Pre-post-test (no control group), France (n of adults in a low-income neighborhood: 199 and 217)	2	1) Social marketing (flyers and informative brochures on physical activity) 3) Networking/partnership (questioning residents on barriers) 4) Environmental change (offering and access to physical activity at community centers; pedestrian orientation paths)	Physical activity behavior (WHO guidelines)	Reduced inequalities (area level)
Cummins et al., 2005 [18]	Quasi-experimental, UK (n of men and women in a deprived community: 493 aged 16+; comparison group: 310)	3	1) Social marketing (advertisement) 4) Environmental change (provision of a new food supermarket)	Fruit and vegetable consumption; self-reported health; psychological health	No difference (area level)
Egan et al., 2016 [19]	Pre-post-test (no control group), UK (n of 14 differentially disadvantaged neighborhoods: 1006)	2	2) Individual or group education (anti-social behavior services/initiatives) 3) Networking/partnership (stakeholders’ consultation including residents) 4) Environmental change (complex housing improvements, demolition and new build)	Self-reported mental and physical health	Reduced inequalities by subgroups (lower income and higher investment groups respectively)
Gans et al., 2018 [29]	Cluster randomized controlled trial, USA (n in 8 intervention sites with low income: 837; 7 control sites: 760)	1	1) Social marketing (motivational campaigns, cooking demonstrations/taste-testing events) 2) Individual or group education (multi-component nutrition education) 4) Environmental change (discount prices, mobile fresh F&V markets)	F&V intake	Reduced inequalities (area level)
Gautam et al., 2014 [34]	Quasi-experimental, New Zealand (n in low-income area: 345; control area: 631)	3	1) Social marketing (biannual information campaign to retailers, wallet card, DVD) 5) Regulatory interventions (controlled purchase operations) 6) Sense of community (social artwork)	Parental and retail supply of tobacco to minors	No difference (area level)
Goodman et al., 2013 [20]	Secondary analysis of census data, UK (n of commuters in 18 intervention cities: 1,266,337; control group: 969,605)	2	2) Individual or group education (e.g. cycle training in schools and colleges) 4) Environmental change (e.g. cycle lanes, cycle parking stands at workplaces)	Cycling/walking to work	Reduced inequalities at area level, but smaller in the most of the deprived areas
Higgerson et al., 2018 [21]	Secondary analysis of two datasets, UK (n of a deprived area: 6160; control area: > 1,5 million for the rest of country)	2	1) Social marketing (considerable promotional activities to raise awareness) 2) Individual or group education (full time equivalent health trainers) 3) Networking/partnership (Healthy Communities Partnership) 4) Environmental change (free access to leisure facilities)	Gym and swim attendances; overall physical activity	Slightly reduced inequalities (area level), greater in the most disadvantaged subgroup
Jongeneel-Grimen et al., 2016 [22]	Quasi-experimental, Netherlands (n of the 40 most deprived districts: 1445; control area: 44,795 for the rest of country)	1	2) Individual or group education (e.g. broad-based primary school activities) 3) Networking/partnership (e.g. action plan tailored to specific local problems) 4) Environmental change (e.g. housing quality, public parks and gardens) 5) Regulatory interventions (e.g. debt assistance and tax reductions, traffic safety) 6) Sense of community (social neighborhood environment)	Mental health	No difference (area level)
Kelaher et al., 2010 [35]	Quasi-experimental, Australia (n of 5 deprived sites: 1479; control sites: 717)	2	2) Individual or group education (e.g. improved employment, learning) 3) Networking/partnership (e.g. action plan with local agencies and residents, Place Manager) 4) Environmental change (e.g. housing, physical environment, increased access to service) 6) Sense of community (increased community pride)	Self-reported health and life satisfaction	No difference at area level, but effective among people being involved in the intervention
Luten et al., 2016 [25]	Quasi-experimental, Netherlands (n of a disadvantaged community: 430 older adults; control group: 213)	2	1) Social marketing (e.g. posters, radio spots, advertorials and press reports, website) 2) Individual or group education (e.g. lifestyle meeting, physical activities for free) 3) Networking/partnership (e.g. promotion by professionals and peers) 4) Environmental change (e.g. healthy eating market, fruit for free)	Physical activity; healthy eating	No differences except for transport-related physical activity
Mohan et al., 2017 [23]	Secondary analysis of data from two panel surveys, UK (n of 36 deprived areas: 596; 3 control areas: 2726)	1	2) Individual or group education (e.g. employability and educational courses) 3) Networking/partnership (e.g. 3-years-action plan by local stakeholders and residents) 4) Environmental change (e.g. housing quality, land developed for green space) 5) Regulatory interventions (e.g. traffic calming schemes, security measures) 6) Sense of community (social neighborhood environment)	Self-rated mental and physical health; life satisfaction; smoking and exercise	No difference or only small trend towards a reduction in inequalities (area level)
O’Loughlin et al., 1999 [30]	Pre-post-test (no control group), Canada (n of a low-income, innercity neighborhood: 819)	3	1) Social marketing (e.g. nutrition campaign, menu-labeling in local restaurants, contests) 2) Individual or group education (e.g. smoking-cessation and nutrition workshops, screening for CVD risk)	Self-reports of smoking, high-fat food consumption, level of physical activity	No difference or only small increase in frequency of cholesterol checkups (area level)
Onion et al., 2019 [31]	Secondary analysis of data from 1971 to 2015, USA (n of one deprived rural area: ~ 22,400; other counties: ~ 994,500)	2	1) Social marketing (e.g. heart healthy menu campaign, brochures on fitness opportunities) 2) Individual or group education (e.g. education and coaching in schools and worksites) 3) Networking/partnership (e.g. lay and professional leadership, health coach collaboration) 4) Environmental change (e.g. new health and fitness center, access to school facilities) 5) Regulatory interventions (e.g. smoke-free recreation areas)	Smoking and mortality rates	Reduced inequalities (area level), but reverted after interventions’ withdrawal
Phillips et al., 2014 [24]	Cluster randomized, UK (n of 20 deprived neighborhoods: 2061 adults; 20 control neighborhoods: 2046)	1	2) Individual or group education (e.g. physical activity sessions, healthy cooking classes) 3) Networking/partnership (e.g. partnerships with local and city-wide organizations) 4) Environmental change (e.g. community gardens and redevelopment of greenspaces, availability of healthy food) 6) Sense of community (intercultural and intergenerational approaches)	Fruit and vegetable consumption; physical activity; mental well-being; social outcomes	No difference (area level)
Raine et al. 2010 [32]	Quasi-experimental, Canada (n of four socioeconomically diverse areas: 4761; control areas: 9775)	1	2) Individual or group education (e.g. leisure activities to encourage people to be active) 3) Networking/partnership (e.g. regular tele-conferences, team meetings) 4) Environmental change (e.g. walking and cycling trails, community gardens) 5) Regulatory interventions (e.g. food security initiatives) 6) Sense of community (promote social inclusion)	Self-perceived health; healthy diet; physical activity, anthropometric; social cohesion	No difference in health outcomes (area level)
Rivera et al., 2004 [38]	Randomized controlled trial, Mexico (n of 347 low-income communities: 578; crossover intervention group: 419)	1	2) Individual or group education (e.g. sessions on nutrition and health education) 4) Environmental change (e.g. food supplements, cash transfers for families associated with medical visits and school attendance)	Height increment and anemia rates in children	Reduced inequalities (area level)
Schulz et al., 2015 [36]	Cluster randomized, USA (n of a low-to-moderate income area: 695 Non-Hispanic Black and Hispanic residents; control group: not reported)	2	2) Individual or group education (training and support lay health promoters, walking group) 3) Networking/partnership (long-standing collaboration among community groups, health service providers, and researchers) 4) Environmental change (e.g. improvements to parks and greenways, safety environment)	Physical activity; CVD risk factors	Reduced inequalities (area level), no difference by SES
Stafford et al., 2014 [25]	Secondary analysis of nation-wide data, UK (n of 39 deprived areas: ~ 17,000; 3 control areas: ~ 3000)	1	2) Individual or group education (e.g. sport or exercise projects, drug/alcohol abuse, food projects, family learning) 3) Networking/partnership (e.g. community commitment, engaging partner agencies in six domains) 4) Environmental change (e.g. housing quality, green/open spaces, access to employment and health services) 5) Regulatory interventions (e.g. Police increase numbers & activity, street lightning, wardens) 6) Sense of community (e.g. sense of community projects)	Self-rated mental and physical health; smoking behavior; social determinants of health	Reduced inequalities in self-rated health and smoking (area level)
Verkleij et al., 2011 [26]	Quasi-experimental, Netherlands (n of intervention region with 50% low-income areas: 3000; reference region: 895)	1	1) Social marketing (e.g. stop-smoking campaign on local television/radio/newspaper, pamphlet distribution) 2) Individual or group education (e.g. computer-tailored nutrition education, supermarket tours) 3) Networking/partnership (e.g. local health committees, public-private collaboration) 4) Environmental change (e.g. food labeling, creating walking and bicycling clubs) 5) Regulatory interventions (e.g. smoke-free areas)	Quality of life (QoL)	No difference at area level, but decrease of mental QoL in subjects with moderate/high SES
White et al., 2016 [27]	Quasi-experimental, UK (n of 35 most deprived areas: 4197; 75 control areas: 6695)	3	2) Individual or group education (e.g. providing teaching assistants, computer skills training) 3) Networking/partnership (e.g. community multiagency partnership boards) 4) Environmental change (e.g. housing maintenance and redeveloping wasteland, installing street lightning, sports equipment) 6) Sense of community (e.g. building community facilities)	Mental health	Reduced inequalities in mental health (area level)
Zapata Moya, Navarro Yáñes, 2017 [28]	Quasi-experimental, Spain (residents of 59 deprived areas: 245,337; 59 control areas: 218,462)	1	4) Environmental change (e.g. re-built houses and buildings, public space, promoting access to health services) 6) Sense of community (e.g. community life facilities)	Preventable and less-preventable mortality	Reduced inequalities in preventable mortality, but not in less-preventable mortality

^a^ 1 = strong, 2 = moderate, 3 = weak study quality (global rating according to EPHPP [39])

With regard to the six types of interventions mentioned above, we found notable differences in the number and combination of these strategies. Sixteen articles explicitly emphasized the component of networking and partnership with local organizations (e.g. sport clubs) and volunteers. Other strategies were rather traditionally shaped, including some types of individual or group education (18 studies), social marketing (11 studies) and/or strategies to promote the “sense of community” (10 studies). Many studies used environmental change or regulatory strategies in specific settings (20 and 8 studies, respectively). Only one of the programs contained elements of all six strategies [16]. Six programs comprised five strategies [22, 23, 25–27, 32], five consisted of four [21, 23, 25, 28, 37], six of three [17, 19, 29, 33, 34, 36] and five of two strategies [18, 20, 28, 30, 36].

Twelve studies aimed at the improvement of health behavior (e.g. physical activity, F&V intake, and smoking), eleven studies examined self-reported mental and physical health, and five studies additionally examined anthropometric outcomes.

### Risk of bias

Included studies were assessed for risk of bias using the “Quality Assessment Tool for Quantitative Studies”, developed by the Effective Public Health Practice Project (EPHPP) [39]. Studies were scored against six criteria (selection bias, study design, confounders, blinding, data collection method, withdrawals and drop-outs), and the number of weak ratings was summed up to give a global quality score. Of the 23 studies reviewed, ten studies were found to be of strong quality (43.8%) [23, 25, 26, 27, 28, 30, 31, 34, 36 39]. Nine studies (39.1%) were moderate in quality [16, 17, 19–21, 23, 32, 34, 37], and four studies (17.4%) were weak in quality [18, 28, 31, 36]. 52.2% of all studies showed poor ratings or could not be evaluated regarding withdrawals and drop-outs. An additional file shows this in more detail [see Additional File 2].

### Impact on health inequality

In the data extraction, we explicitly aimed to identify studies which were carried out in socially disadvantaged communities and/or conducted analyses of outcome measures by subgroups of SES such as income, education, and/or occupation. Out of the 23 studies that met our inclusion criteria, 13 (56.5%) reported reductions of health inequalities in the entire (deprived) neighborhood or community [16, 17, 19–21, 25, 28–30, 32, 34, 35, 38]. However, this included one study in which there was little impact on health equity, but even more negative effects [16]. In two further studies (8.7%), despite the lack of evidence at the level of the entire community, beneficial effects could be found in persons who belonged to the most disadvantaged subgroup within the area [27] or who were particularly exposed to the intervention [37].

Among the remaining eight (34.8%) studies, no differences were found between intervention and control areas [18, 23–26, 32, 35]. Only in one differential sub-analysis a small intervention effect was found among those with a low educational level for energy intake and walking/bicycling [40]. No other studies except the two mentioned had analyzed the outcomes by socio-economic subgroups within an area to search for a specific social gradient in health. These results seem to be rather sobering and inconsistent, involving both large-scale (e.g. [25]) or smaller (e.g. [23]) programs with a very diverse mix of intervention strategies and outcome parameters. An important point in this context, however, is that there was, with one exception [16], no evidence that community-based programs included in our review resulted in any widening of health disparities (“intervention-generated inequalities” or IGIs) at community-wide level.

## Discussion

Our review shows that complex community-based interventions can contribute to reducing socio-economic inequalities in health behavior and health status outcomes, or at least do not increase inequalities, respectively. The findings suggest that multi-level, multi-component interventions can be effective due to synergistic effects between multiple intervention components; besides this, complex interventions reside in the degree of flexibility or tailoring of the intervention permitted [41, 42]. Our results are also congruent with existing summaries of what is known about the effect of different categories of interventions on inequalities, particularly “upstream” interventions in the wider social (policy level) determinants: e.g. reducing price barriers, fiscal interventions, and housing [1, 3]. Similarly, there is suggestive evidence that large, long-lasting urban renewal programs may positively affect physical and mental health, but the actual effects may be small [28, 29].

However, there was no consistent evidence to support the impact of such interventions in reducing the social health gap *within* an intervention population. To achieve an equity impact, healthy lifestyle interventions as well as activities to create a healthier environment need to be delivered in an adequate “dose” to stimulate or support health changes [9, 43]. Thus, it has to be kept in mind that both the reach (significant proportion of the population being affected) and the intensity (frequency and duration of intervention components) of a neighborhood program are important. In many community intervention studies there is a high demand for process evaluation in order to assess the degree to which the intervention was implemented and met the dose as planned [41]. For example, we found some evidence for a dose-response association between length of residence in a regeneration area and a `higher` level of investment during the study period, and improvements in mental and physical health respectively [19, 28, 44].

White et al. described additional factors in the implementation of an intervention which may impact upon differential effectiveness by SES, including stages of the provision of, and responses to a health intervention [8]. Compliance may be higher among more advantaged groups due to better access to resources such as finance, time and coping skills. According to Roger’s theory of diffusion, interventions may therefore (at different times) be more likely to be taken up by those persons who are of higher SES and are more likely to widen the health gap [8, 45]. Conversely, the less educated or affluent groups are less able to access the intervention, understand it or engage in it. For this reason, interventions that are provided in the same way to all residents may result in differential outcomes. This is likely another characteristic of complex interventions that may widen inequality. Tailoring interventions need new ways especially for low socio-economic groups [46, 47].

Many community-based programs reviewed here relied on participation as a means of community involvement in the program planning and implementation. Thus, intervention-generated inequalities could have taken place, for example, when a community survey is used to assess the need for intervention. Socio-economic variations in response rates may lead to underestimation of need in the most socially disadvantaged groups [48]. Similarly, low SES groups are often less well represented in follow-up studies, leading to an attrition bias in the assessment of outcomes [49]. To address imbalances in power between socio-economic groups, the interventions need flexibility to ensure that they will be suited to the needs and perceptions of specific sub-groups, thus increasing participation and intervention effectiveness [41]. More community involvement increases the amount of time needed for intervention planning and implementation, and may have implications for the cost of such studies. In the long term, however, application of these methods is likely to contribute to improved intervention effectiveness and equity [41, 50].

### Limitations of the review

There are a number of limitations concerning the present review. Initial searches of databases identified several thousand references, but the small number of eligible studies suggests that few health outcome evaluations of complex community-based interventions have been published in peer-reviewed journals in the last 20 years. Thus, the review described here is possibly not exhaustive and does not cover studies from low income countries. Our search strategy may not have revealed a complete list of all studies describing intervention effects by SES due to limitations of the Pubmed and PsycINFO databases. Single interventions and/or sub-settings (e.g. school, kindergarten) were excluded. Finally, the vast majority of studies identified targeted on effects at a low-SES population level, and did not explore differential effects on inequalities by SES sub-groups.

## Conclusions

Despite the limitations described above, our review suggests that multiple community-based interventions in health promotion and prevention may contribute to reducing inequalities at area level, but their potential is not fully realized. Thus, based on this review, no final recommendations can be made for national policies. However, there are national and international initiatives that support the notion that health inequalities can be reduced by such interventions. For example, the German cooperation-network “Equity in Health” [51] mentions the community as an important setting for health promotion and defines criteria for good practice of community-based health promotion activities. There are similar initiatives in Europe highlighting the importance of complex health promotion interventions on the community level to reduce health inequalities [52]. Further studies should examine in more detail whether there is a change of health inequalities within an intervention area which affects the overall change in population health. Likewise, the results highlight the importance of including at least some measures of process evaluation in order to appropriately assess the benefits of these interventions on equity in health.

## Data Availability

Not applicable.
